# Multiplex ligation-dependent probe amplification and array comparative genomic hybridization analyses for prenatal diagnosis of cytogenomic abnormalities

**DOI:** 10.1186/s13039-014-0084-5

**Published:** 2014-12-09

**Authors:** Zhiyong Xu, Qian Geng, Fuwei Luo, Fang Xu, Peining Li, Jiansheng Xie

**Affiliations:** Shenzhen Maternity and Child Healthcare Hospital, Shenzhen, Guangdong China; Department of Genetics, Yale University School of Medicine, New Haven, CT USA

**Keywords:** Multiplex ligation-dependent probe amplification (MLPA), Array comparative genomic hybridization (aCGH), Prenatal diagnosis, Cytogenomic abnormalities

## Abstract

**Background:**

The aims of this study were to evaluate the clinical utility of multiplex ligation-dependent probe amplification (MLPA) and array comparative genomic hybridization (aCGH) analyses on prenatal cases and to review prenatal ultrasound findings of cytogenomic syndromes.

**Results:**

Of the 54 prenatal cases analyzed, cytogenomic abnormalities were characterized in 14 cases. In four fetuses with abnormal ultrasound findings, a 40.701 Mb duplication of 8q22.3-q24.3 and a 23.839 Mb deletion of 7q33-q36.3 derived from a paternal balanced translocation, a de novo 13.062 Mb deletion of 11q24.1-q25 for Jacobsen syndrome, a de novo 19.971 Mb deletion of 7q11.23-q21.3 for type 1 split-hand/foot malformation (SHFM1), and a de novo 28.909 Mb duplication of 3q21.1-q25.1 were detected. A 699.8 Kb deletion at 5p15.33 for Cri du Chat syndrome was confirmed in a fetus with abnormal MLPA result. A fetus with abnormal maternal screening was detected with a de novo distal 1.747 Mb duplication at 2q37.1-q37.2 and a 6.664 Mb deletion at 2q37.2-q37.3. Of the eight cases referred by history of spontaneous abortions, derivative chromosomes 11 from paternal carriers of a balanced 8q/11q and a 10q/11q translocation were noted in two cases, simple aneuploids of trisomy 2 and trisomy 21 were seen in three cases, and compound aneuploids of two or three chromosomes were found in three cases. Post-test genetic counseling was performed with detailed genomic information and well characterized postnatal syndromic features.

**Conclusions:**

These results demonstrated that coupling MLPA screening and aCGH analysis are a cost-effective approach to detect cytogenomic abnormalities in a prenatal setting. The aCGH analysis provided not only genomic maps of breakpoints and gene content of imbalanced regions but also better inference of related phenotypes for genetic counseling. Prenatal ultrasound findings reported in the literature for Jacobsen syndrome, SHFM and Cri du Chat syndrome were summarized for use as diagnostic references.

## Background

Prenatal genetic diagnosis has been driven by innovative technologies and accumulated clinical knowledge. Since late 1970s, invasive amniocentesis of amniotic fluid (AF) and chorionic villus sampling (CVS) procedures, non-invasive maternal serum screening, and high resolution ultrasound examination have been introduced and become the standard of care in prenatal diagnosis. The major clinical indications for prenatal diagnosis include advanced maternal age (AMA) for increased risk of Down syndrome, abnormal maternal serum screening (aMSS), abnormal ultrasound (aUS) findings, family history (FH) of chromosomal or genetic disorders, history of spontaneous abortion (hSAB), and recently integrated maternal serum fetal DNA sequencing for aneuploidy screening [1-4]. In a retrospective study, advances in maternal serum screening and ultrasonography have resulted in more than 50% decline in AF and CVS procedures, while the diagnostic yield for chromosomal abnormalities increased from 2% in 1991 to 7% in 2002 [2]. A recent report of prenatal cases from 2007 to 2009 showed an abnormality detection rate of 10.2% for numerical chromosomal abnormalities and 1.9% for structural chromosomal abnormalities [3]. However, routine prenatal cytogenetic analysis has two obvious limitations: the low analytical resolution of about 5–10 megabase (Mb) by the Giemsa banding and the long turn-around time due to the in vitro cell culture procedures. Various DNA-based molecular approaches have been introduced to provide rapid prenatal screening and diagnosis. Fluorescence in situ hybridization (FISH) using labeled DNA probes in the size of 100–800 kilobase (Kb) has enhanced the analytical resolution and allowed rapid screening of locus-specific numerical aberrations. A multiplex FISH panel with differentially labeled probes was developed for prenatal screening of common aneuploidies of chromosomes X, Y, 13, 18 and 21 [5]. Quantitative fluorescence-polymerase chain reaction (QF-PCR) had been introduced for prenatal diagnosis of fetal aneuploids [6,7]. Multiplex ligation-dependent probe amplification (MLPA) was validated as a rapid, cost-effective and multi-allelic approach for prenatal screening of common aneuploids, recurrent genomic disorders and subtelomeric imbalances [8-11]. For pediatric patients with intellectual and developmental disabilities, genome-wide array comparative genomic hybridization (aCGH) using synthesized oligonucleotides has been validated and recommended as first tier genetic testing [12-14]. Extended application of this aCGH analysis on prenatal diagnosis has been effective in defining gene content of chromosomal imbalances and in detecting genomic disorders and cryptic aberrations [15-17]. However, there are technical challenges and clinical concerns for rapid replacement of prenatal cytogenetics with genomic analysis [18]. In the technical front, proper DNA extraction avoiding maternal cell contamination, cautious on confined-placental mosaicism, and timely confirmatory and follow up parental analyses need to be considered. While in the clinical part, the benefit, limitations and possible outcomes should be discussed in pre- and post-testing genetic consulting. In this study, we present an approach coupling MLPA and aCGH analyses for prenatal detection of cytogenomic abnormalities. Fourteen prenatal cases with chromosomal rearrangements and submicroscopic abnormalities were defined and genetic counseling was conducted with more detailed genomic characteristics. We also reviewed prenatal ultrasound findings reported in literature for Jacobsen syndrome, SHFM1 and Cri du Chat syndrome.

## Results

**Figure 1:**
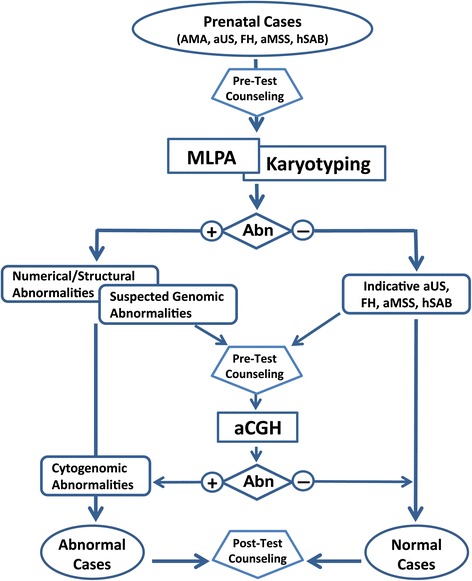
**A workflow showing an integrated MLPA screening, Karyotyping and aCGH analysis for prenatal diagnosis of cytogenomic abnormalities.** AMA, advanced maternal age; FH, family history of genetic disorders; aUS, abnormal ultrasound findings; hSAB, history of spontaneous abortion.

The workflow of MLPA screening and routine karyotyping for all prenatal referrals and aCGH analysis on selected cases was shown in Figure 1. A total of 54 patients (18 cases with aUS, 13 by FH, 12 with hSAB, four with aMSS, four with hSAB and FH, one with aUS and aMSS, one with aUS and FH, and one with normal US/aMSS/FH but abnormal MLPA result) were elected to have further aCGH analysis. The karyotyping, MLPA screening and aCGH results of 14 abnormal cases and participated parents are summarized in Table 1. The patient history, detailed prenatal clinical findings and cytogenomic results for each case are described as follows:

**Table 1:** Summary of Prenatal Clinical Features and Abonormalities Detected by Karyotyping, MPLA and aCGH*

**Case no. [sample]**	**Clinical indication**	**Karyotype**	**MLPA (Region-Specific Assay, rsa)**	**aCGH findings (hg18)**	**Parental study**	**Follow-up**
1 [AF]	aUS	46,XY,der(7)t(7;8) (q33;q22.3)pat	rsa 8q24.3(P036)×3,7q36.3(P036)×1	arr 8q22.3q24.3(105,474,143-146,175,291)x3, 7q33q36.3(134,972,470-158,811,468) x1	46,XY,t(7;8)(q33;q22.3)	Terminated
2 [AF]	aUS	46,XX,del(11)(q23.3)dn	rsa 11q25(P036)×1	arr 11q24.1q25(121,377,483-134,439,406)x1	Normal	Terminated
3 [AF]	aUS	46,XY,del(7)( q11.23q21.3)dn	Normal	arr 7q11.23q21.3(76,539,754-96,510,594)x1	Normal	Terminated
4 [AF]	aUS	46,XX,dup(3)(q21.1q25.1)	Normal	arr 3q21.1q25.1(124,410,610-153,319,948)x3	Normal	Terminated
5 [AF]	MLPA	46,XX	rsa 5p15(P245)×1	arr 5p15.33(711,361-1,411,206)x1	Normal	Term delivery
6 [AF]	aMSS	46,XY,del(2)(q37)dn	rsa 2q37.3(P036)×1	arr 2q37.2(234,210,736-235,957,912)x3, 2q37.3(236,012,851-242,677,269)x1	Normal	Terminated
7 [CVS]	hSAB	46,XY,der(11)t(8;11) (q24.1;q23.3)pat	rsa 8q24.3(P036)×3,11q25(P036)×1	arr 8q24.13q24.3(125,657,117-146,265,147)x3, 11q23.3q25(116,350,093-134,432,412)x1	46,XY, t(8;11)(q24.1;q23.3)	Terminated
8 [CVS]	hSAB	46,XY,der(11)t(10;11) (q26.1;q24.1)pat	rsa 10q26.3(P036)×3,11q25(P036)×1	arr 10q26.13q26.3(126,217,300-135,284,309)x3, 11q24.1q25(122,322,510-134,432,465)x1	46,XY,t(10,11)(q24.1;q26.1)	Terminated
9 [CVS]	hSAB	N/A	rsa X(P095)x2,Y(P095)x1, 21(P095)x3,22p13(P036)×3, 22q13.3(P036)×3	arr(X)x2,(Y)x1,(21,22)x3	Normal	SAB
10 [CVS]	hSAB	N/A	rsa X(P095)x2,Y(P095)x1, 10p15(P036)×3,10q26.3(P036)×3	arr(X)x2,(Y)x1,(10)x3	Normal	SAB
11 [CVS]	hSAB	N/A	rsa 21(P095)x3,8p23.3(P036)×3, 8q24.3(P036)×3	arr(8,21)x3	Normal	SAB
12 [CVS]	hSAB	N/A	rsa 2p25.3(P036)×3,2q37.3(P036)×3	arr(2)x3	Normal	SAB
13 [CVS]	hSAB	N/A	rsa 2p25.3(P036)×3,2q37.3(P036)×3	arr(2)x3	Normal	SAB
14 [CVS]	hSAB	N/A	rsa 21(P095)x3	arr(21)x3	Normal	SAB

*CV, chorionic villus; AF, amniotic fluid; aMSS, abonormal maternal serum screen; aUS, abnormal ultrasound; hSAB, history of spontaneous abortions; N/A, not applied.

### Case 1

Amniocentesis was performed because of multiple malformations detected at 12 weeks of gestation. The ultrasonographic examination revealed fetal growth retardation, bilateral cleft lip and palate, right-sided aortic arch, multiple cystic hypoplasias. MLPA analysis indicated a subtelomeric duplication of 8q and a subtelomeric deletion of 7q. Chromosome analysis performed on cultured amniocytes showed a derivative chromosome 7 from a translocation between chromosomal bands 7q33 and 8q22.3. Further aCGH analysis revealed a 40.701 Mb duplication of 8q22.3-q24.3 (105,474,143-146,175,291) including genes from *DPYS* to *ZNF16* and a 23.839 Mb deletion of 7q33-q36.3 (134,972,470-158,811,468) including genes from *NUP205* to *VIPR2*. Follow up chromosome analysis on both parents detected the father as a carrier of a balanced translocation of 7q33/8q22.3.

### Case 2

**Figure 2:**
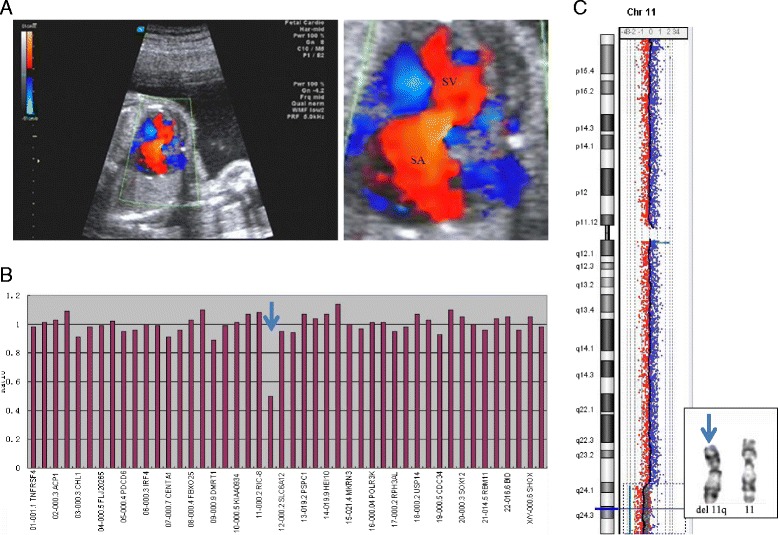
**Ultrasound findings and chromosomal abnormality characterized in case 2. A**. A four chamber view shows single ventricle (SV), single atrium (SA) and common atrioventricular valve. A color Doppler flow image shows the bloodstream of atrioventricular valve was flowed from SV to SA. **B**. MLPA shows an 11q deletion (arrow). **C**. aCGH reveals a 13.062 Mb deletion of 11q24.1q25 (inset, arrow points to the distal deleted chromosome 11).

Amniocentesis was performed because congenital heart defect was detected at 24 weeks of gestation. A four chamber view showed single ventricle, single atrium and common atrioventricular valve (Figure 2A). MLPA analysis detected a subtelomeric deletion of 11q (Figure 2B). Further aCGH analysis of DNA from cultured amniocytes revealed a 13.062 Mb deletion of 11q24.1-q25 (121,377,483-134,439,406) including genes from BC089451 to AK130852; chromosome analysis performed on cultured amniocytes showed an 11q23.3 deletion (Figure 2C). Parental chromosome analysis showed normal results. The parents elected pregnancy termination.

### Case 3

**Figure 3:**
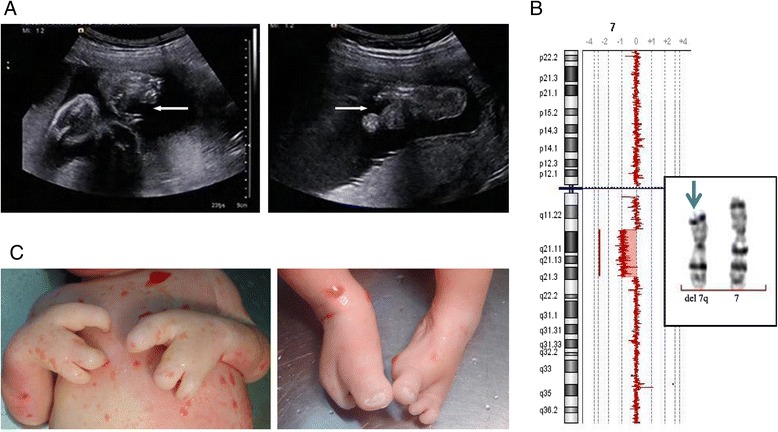
**Ultrasound findings and chromosomal abnormality characterized in case 3. A**. The ultrasonography shows split-hand split-foot malformation. **B**. aCGH reveals a 19.971 Mb deletion of 7q11.23q21.3 (inset, arrow points to the interstitial deleted chromosome 7). **C**. The induced abortion shows split-hand split-foot malformation.

Amniocentesis was performed at 28 weeks gestation because of ultrasound detected anomalies of split-hand split-foot malformation and multiple umbilical cord cysts (Figure 3A). MLPA performed on DNA extracted from non-cultured aminiocytes showed normal result. Further aCGH analysis of DNA from cultured amniocytes revealed a 19.971 Mb deletion of 7q11.23-q21.3 (76,539,754-96,510,594) including genes from *CCDC146* to *DLX5*; chromosome analysis performed on cultured amniocytes confirmed the 7q11.23-21.3 deletion (Figure 3B). Parental chromosome analysis showed normal results. The parents elected pregnancy termination. The postmortem examination showed the deformities of the hands and feet (Figure 3C).

### Case 4

Amniocentesis was performed at 23 weeks gestation because of ultrasound detected anomalies of an increased nuchal translucency (NT) and fetal growth retardation. MLPA performed on DNA extracted from non-cultured aminiocytes showed normal result. Further aCGH analysis of DNA from cultured amniocytes revealed a 28.909 Mb duplication of 3q21.1-q25.1 (124,410,610-153,319,948) including genes from *SEC22A* to *SUCNR1*. Chromosome analysis performed on cultured amniocytes confirmed the 3q21.1-q25.1 duplication. Parental chromosome analysis showed normal results. The parents elected pregnancy termination.

### Case 5

Amniocentesis was performed because of abnormal MLPA result of a deletion in the Cri du Chat region. Ultrasound examination at 18 weeks gestation found a normal result. Chromosome analysis of cultured amniocytes showed a normal result. Follow-up aCGH analysis revealed a 699.8 Kb deletion at 5p15.33 (711,361-1,411,206) including genes *TPPP*, *ZDHHC11*, *BRD9*, *TRIP13*, *NKD2*, *SLC12A7*, *SLCA19*, *SLC6A18*, *TERT* and *CLPTM1L*. The parents decided to continue the pregnancy and the fetus was delivery at term. Follow up communication to the mother suggested that the girl appeared normal at age of two years.

### Case 6

Amniocentesis was performed at 22 gestational weeks because of the abnormal second trimester screening result suggesting an increased risk for Down’s syndrome. MLPA performed on DNA extracted from non-cultured aminiocytes detected a subtelomeric deletion of 2q and chromosome analysis performed on cultured amniocytes revealed a 2q37 deletion. Further aCGH analysis of DNA from cultured amniocytes revealed a 1.747 Mb duplication at 2q37.1-q37.2 (234,210,736-235,957,912) including genes from *UGT1A8* to *SH3BP4* and a 6.664 Mb deletion at 2q37.2-37.3 (236,012,851-242,677,269) including genes from *AGAP1* to *PDCD1*. Parental chromosome analysis showed normal results. The parents elected pregnancy termination.

### Case 7

**Figure 4:**
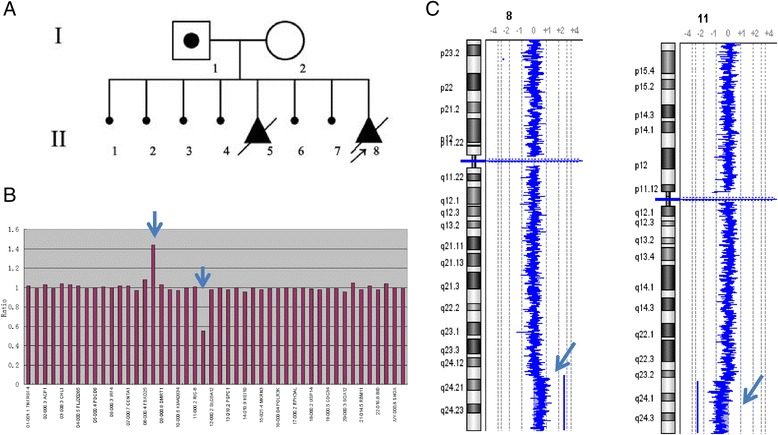
**Chromosomal structural rearrangement characterized in case 6. A**. Pedigree shows the father (I-1) as a carrier for an 8q/11q balanced translocation and the tested fetus (II-8) and previous abortions likely resulting from unbalanced recombinants. **B**. MLPA shows an 8q duplication and an 11q deletion (arrows). **C**. aCGH reveals a 20.608 Mb duplication of 8q24.13q24.3 and an 18.082 Mb deletion of 11q23.3q25 (arrows).

CVS was performed because of the hSAB and absence of fetal heart by ultrasound examination at 8 weeks gestation. The woman had aborted seven times, including six times of fetal anomalies in earlier gestational period and one induced labor due to the malformation at 28 gestational weeks (Figure 4A). MLPA analysis performed on DNA extracted directly from CVS detected a subtelomeric duplication of 8q and a subtelomeric deletion of 11q (Figure 4B). Further analysis using aCGH revealed a 20.608 Mb duplication of 8q24.13-q24.3 (125,657,117-146,265,147) including genes from *MTSS1* to C8orf33 and an 18.082 Mb deletion of 11q23.3-q25 (116,350,093-134,432,412) including genes from KIAA0999 to *B3GAT1* (Figure 4C). Chromosome analysis on cultured fibroblasts from the villi detected the derivative chromosome 11; follow up parental chromosome analysis showed a normal female karyotype in the mother and a balanced translocation between chromosomal bands 8q24.13 and 11q23.5 in the father.

### Case 8

CVS was performed because of hSAB and absence of fetal heart by ultrasound examination at 8 weeks gestation. MLPA analysis indicated a subtelomeric duplication of 10q and a subtelomeric deletion of 11q. Further aCGH analysis revealed a 9.067 Mb duplication of 10q26.13-q26.3 (126,217,300-135,284,309) including genes from *LHPP* to *SYCE1* and a 12.110 Mb deletion of 11q24.1-q25 (122,322,510-134,432,465) including genes from C11orf63 to *B3GAT*. Follow up parental chromosome analysis found that the father is a carrier for a balanced translocation between chromosomal bands 10q26 and 11q24.

### Cases 9–14

CVS was performed because of hSAB for the six patients. MLPA analyses detected XXY sex chromosome and three copies of chromosomes 21 and 22 in patient 9, XXY sex chromosome and three copies of chromosome 10 in patient 10, three copies of chromosomes 8 and 21 in patient 11, three copies of chromosome 2 in patients 12 and 13, and three copies of chromosome 21 in patient 14. Follow up aCGH analyses confirmed the MLPA results for all six patients and ruled out other submicroscopic abnormalities. Parental chromosome analyses on all of them found normal results.

## Discussion

Prenatal genetic counseling for detected cytogenomic abnormalities involves first the assessment of fetal viability and second the prediction of severity of phenotype from the family history and the clinical evidence in the literature. Of the 14 cases with defined cytogenomic abnormalities, four had aUS findings, one was noted from abnormal MLPA result, one was due to aMSS and eight were referred by hSAB. Cytogenomic abnormalities detected in four cases with aUS findings allowed further evaluation of genotype-phenotype correlation. A large 40.701 Mb duplication of 8q22.3-q24.3 and a large 23.839 Mb deletion of 7q33-q36.3 were found in case 1. The large segmental imbalances likely explain the observed malformations. A large 13.062 Mb deletion of 11q24.1-q25 was found in case 2, which is diagnostic for Jacobsen syndrome (OMIM#147791). A recent study found that targeted deletion of the *ETS1* gene results in ventricular septal defects and abnormal ventricular morphology in mice [19]. The *ETS1* gene at 11q24.3 is within the large 11q deletion of cases 2 and its haploinsufficiency likely explains the prenatally detected heart defects. The 7q11.23-q21.3 deletion in case 3 is diagnostic for the prenatally detected SHFM1 (OMIM#183600). In case 5, aCGH analysis revealed a 699.8 Kb deletion including the *TERT* gene (telomerase reverse transcriptase) at 5p15.33. This is a submicroscopic deletion undetectable by routine chromosome analysis. The *TERT* gene has been reported as a candidate gene for Cri du Chat syndrome (OMIM#123450); the most important clinical features are a high-pitched cat-like cry, distinct facial dysmorphism, microcephaly, severe psychomotor and mental retardation [20,21]. However, the normal prenatal ultrasound finding and appear normal development at age of two years in case 5 may represent a mild phenotype. This case raises concern on prenatal genetic counseling of known microdeletion syndrome. A longitudinal study on this case is recommended to the parents. Prenatal ultrasound findings reported in literature for Jacobsen syndrome [22-26], SHFM [27-30] and Cri du Chat syndrome [31-36] are reviewed and summarized in Table 2. For Jacobsen syndrome, prenatal ultrasound showed heterogeneous findings but hypoplastic heart defect or ventricular anomalies have been observed at gestational age from 20 ~ 28 weeks; and a distal deletion of 11q23 was found in all cases (Table 2) [22-26]. For limb deficiencies, an earlier report of prenatal diagnosis of ectrodactyly observed the ‘lobster claw’ anomaly in two cases and also reviewed three case reports in the literature. However, the genetic etiology for these cases was unknown [37]. The EUROSCAN study of limb reduction deficiencies found that limb reduction deficiencies were seen in 0.035% of pregnancies. Of the 54 cases studied by chromosome analysis, about 30% (16/54) were detected with a chromosomal abnormality including trisomies 18 and 21, Klinefelter syndrome and a 7q deletion [38]. This EUROSCAN result indicated that the limb deficiencies could be an indicator for chromosomal abnormalities. Review reports of prenatal ultrasound findings for SHFM noted the detection of bilateral or unilateral split hand foot malformations at gestational age from 11–29 weeks; a *TP63* gene mutation, a 22q11.2 deletion for DiGeorge syndrome and a 7q31 deletion was noted in three cases and the genetic etiology for ten cases remained unknown (Table 2) [27-30]. For Cri du Chat syndrome caused by a 5p deletion, pleural effusion, cerebral ventricular anomalies, hypoplastic or absent of nasal bone were recurrent aUS findings but a mild borderline microcephaly was seen in one mosaic case [31-36]. These observations demonstrated that indicative fetal anomalies for these cytogenomic syndromes could be identified by ultrasound examination although genetic heterogeneity for SHFM and Cri du Chat should be taken into consideration. For all these abnormal cases, the post-test counseling with detailed genomic information helps both the counselors with better phenotype prediction and the parents for an informed decision.

**Table 2:** **Abnormal ultrasound findings in Jacobsen syndrome, SHFM and Cri du Chat sydnrome**

**Syndrome**	**Gastational age**	**Abnormal ultrasound findings**	**Etiology**	**References**
**Jacobsen**	20 wks	Nuchal thickening (NT)	del(11)(q23)	McClelland, 1998 [22]
	20 wks	Unilateral duplex renal system, pyelectasis, orofacial clefts.	del(11)(q23)	Chen, 2001 [23]
	20 wks	Fetal biometry ~22 wks, short femurs and humeri, overlapping toes	del11)(q24.2)	Chen, 2004 (2 cases) [24]
	18 wks	Unremarkable	del(11)(q24.1)	
	20 wks	Cerebral ventricular dilatation	del(11)(q23)	Bohem, 2006 [25]
	28 wks	Hydronephrosis, hypoplastic lefft hear syndrome, 'keel-shaped' skull of trigonocepholy.	del(11)(q23)	Foley, 2007 [26]
	24 wks	Single ventricle, single atrium, atrioventricular valve	del(11)(q24.1)	This report (case#2)
**SHFM**	12-15-19 wks	NT 1.0 mm, single digit hand, two-digit split-foot.	Unknown	Haak, 2001 [27]
	11-16 wks	Irregular shaped hands with missing digits, clawlike feet.	Unknown	Ram, 2009 [28]
	19 wks	Bilateral Cleft Lip/palate, median clefts of both hands and feet, oligodactyly.	c.598A>G TP63 gene	Simonazzi, 2012 [29]
	15-29 wks	7 bilateral anomalies (3 both hands and foot, 2 only in hands and 2 only foot) and 3 unilateral anomalies in hands.	22q11.2 del, 7q31 del, 8 unknown	Lu, 2014 (10 cases) [30]
	28 wks	Split hand/split foot, umbilical cord cysts.	del(7)(q11.23q21.3)	This report (case#3)
**Cri du Chat**	16 wks	Pleural effusion, ascites, skin edema, hypoplastic cerebellum, large ventricular septal defect, cystic lesion in nuchal skin edema.	5p-	Aoki, 1999 [31]
	15+2 wks	Moderate biparietal cerebral ventriculomegaly	del(5)(p15)	Stefanou, 2002 [32]
	19 wks	Boderline microcephaly	mos del(5)(p15.1)	Chen, 2004 [33]
	18+6 wks	Encephalocele	del(5)(p13)	Bakkum 2005 [34]
	21+3 wks	Absent nasal bone	del(5)(p15.1)	Sherer, 2006 [35]
	13 wks	NT 1.6 mm, hypoplastic nasal bone (1.8 mm)	del(5)(p14)	Teoh, 2009 [36]
	18 wks	Normal	del(5)(p15.33p15.33)	This report (case#5)

The clinical impact from recognized compound segmental deletion and duplication in cases 1, 6, 7 and 8 should be interpreted with caution in a prenatal setting. The detected paternal carrier of a balanced translocation in cases 7 and 8 provides genetic explanation for the recurrent spontaneous abortions. Actually, parental chromosome analyses for case 7 were performed three times in other hospitals; the reciprocal translocation in the father, denoted as t(10;11)(q26.13;q24.1), was missed due to the similar size and light G-band pattern of the translocated segments. This case indicated that genomic characterization of prenatal segmental imbalances could facilitate karyotyping recognition of hidden subtelomeric rearrangements. Interestingly both cases 7 and 8 showed absence of fetal heart at ultrasound examination which could be explained partially by the shared 11q deletion for Jacobsen syndrome and partially by compound effect from other segmental imbalances. The other six patients (cases 9–14) with hSAB were detected with simple and compound aneuploids but no other pathogenic copy number variants. Parental chromosome analyses performed on these six families showed normal findings.

Technically, FISH and QF-PCR have been used for rapid screening of prenatal aneuploidy but these methods were limited to a few chromosomal loci [5-7]. MLPA using aneuploidy probe mix (P095), subtelomeric probe mixes (P036 and P070) and microdeletion syndromes probe mix (P245) has been a robust method for rapid prenatal screening of aneuploidy, unbalanced subtelomeric rearrangements and recurrent microdeletions. The turn-around-time of MLPA screening is usually within one to three days. However, MLPA screening lacks the genome-wide coverage and cannot detect copy number changes outside the represented regions. For example, MLPA missed the deletion of 7q11.23-q21.3 in case 3 and duplication of 3q21.1-q25.1 in case 4. In cases 6, MLPA detected the 2q deletion but missed the coexisted 2q duplication. Therefore, MLPA should be used as a rapid and cost-effective screening for common aneuploids, subtelomeric rearrangements and known genomic disorders. Follow up genome-wide aCGH analysis is strongly recommended to define genomic coordinates for MLPA and karyotype detected abnormalities and to rule out other copy number aberrations. Recently, genomic SNP array has been proposed as a gold standard for prenatal diagnosis of fetal ultrasound abnormalities [39], but rapid replacement of prenatal cytogenetic analysis raised serious concerns [18]. One major concern is the detection of variants of unknown significance (VUS), which can complicate the genetic counseling and add unnecessary anxiety to the pregnant women. Of the 54 prenatal cases analyzed by aCGH in this report, no VUS was detected. The other concern is the high cost of aCGH analysis, which likely explains the low acceptance rate for further aCGH analysis in our experience. It is worth noted that aCGH analysis also has its limitations in the detection of polyploidy, balanced chromosomal rearrangements, inversions and low level mosaicism. Although routine chromosome analysis can neither provide the genomic coordinates for chromosomal abnormalities nor detect the submicroscopic copy number changes, karyotyping is still the gold standard for prenatal diagnosis of chromosome aneuploids and structural rearrangements.

## Conclusions

In summary, aCGH is effective in characterizing chromosomal and submicroscopic imbalances. It has been estimated that approximately 10% of pregnancies with ultrasound-detected structural anomalies and normal cytogenetic findings had genomic abnormalities, and 30% of these abnormalities were recurrent genomic disorders [18]. The workflow of MLPA, karyotyping and aCGH analysis as shown in Figure 1 represents a robust and cost-effective approach for prenatal diagnosis of chromosomal aneuploids, subtelomeric rearrangements and genomic imbalances. The MLPA screening results guide focused chromosome analysis of aneuploids and subtelomeric rearrangements and aCGH analysis for fine mapping of breakpoints and gene content of imbalanced regions. Further aCGH analysis on prenatal cases with indicative clinical findings can help to detect or rule out other genomic imbalances. This integrated approach can significantly reduce the number of cases for aCGH and thus reduce the overall cost of prenatal diagnosis.

## Methods

### Case selection

For the past two years, of the 2509 cases analyzed by karyotyping and MLPA, numerical chromosomal and structural abnormalities were noted in 106 (4.2%) and 102 cases (4.1%), respectively. Of cases with structural chromosomal abnormalities detected by karyotyping, suspected genomic abnormalities by MLPA, and indicative findings of aUS, aMSS, FH and hSAB, pre-test genetic counseling with detailed information about the advantages and limitations of aCGH was provided. Post-test genetic counseling for cases with abnormal aCGH results was provided with detailed genomic findings and evidence-based interpretation. This study was reviewed and approved by the Ethics Committee of Shenzhen Maternity and Child Healthcare Hospital and participated patients also provided written informed consent.

### Karyotyping and MLPA analysis

Chromosome analysis was performed on G-band metaphases prepared from cultured cells of amniotic fluid (AF), chorionic villus samples (CVS), and peripheral blood lymphocytes according to the laboratory’s standard protocols. Twenty metaphases were examined for each sample. Genomic DNA was extracted from AF and CVS using the chelex-100 (Bio-Rad Laboratories Inc, Hercules, CA, USA). The DNA concentration was measured using the NanoDrop spectrophotometer (ND-2000, Thermo Fisher Scientific Inc., Waltham, MA, USA). For each MLPA assay, 100 ng of DNA was used following the manufacturer’s protocols. The MLPA assays included the SALSA MLPA P095 aneuploidy probe mix for the detection of aberrant copy number of human chromosomes 13, 18, 21, X and Y, the P036/P070 human subtelomeric probe mix for the detection of deletions and duplications of subtelomeric region of each chromosome, the P245 microdeletion probe mix for 22 regions of 1p36 (OMIM#607872), 2p16 (OMIM#612513), 2q23.1 (OMIM#156200), 2q33 (OMIM#612313), 3q29 (OMIM#609425), 4p16.3 (OMIM#194190), 5p15.2 (OMIM #123450), 5q35 (OMIM#117550), 7q11.23 (OMIM#194050), 8q24.1 (OMIM#150230), 9q22.3, 10p14 (OMIM#601362), 15q11.2 (OMIM#176270, OMIM#105830), 15q24 (OMIM#613406), 16p13.3 (OMIM#180849), 17p13.3 (OMIM#607432, OMIM#247200) 17p11.2 (OMIM#182290), 17q11.2 (OMIM#162200), 17q21 (OMIM#610443), 22q11.2 (OMIM#188400), 22q13.3 (OMIM#606232) and Xq28 (OMIM#312750).

### Array comparative genomic hybridization (aCGH)

For each sample, 2 μg of genomic DNA was prepared following the manufacturers protocol for the Agilent Human Genome CGH microarray 60 K kit (SurePrint G3 Human CGH 8x60K Microarray Kit) or 180 K kit (SurePrint G3 Human CGH 4×180K Microarray Kit) (Agilent Technologies Inc, Santa Clara, CA, USA). This validated aCGH procedure can achieve 99% sensitivity and 99% specificity with an analytical resolution determined by a sliding window of five to seven contiguous oligonucleotides [12]. The base pair designation was based on the NCBI36/hg18 assembly of the UCSC Human Genome browser (http://genome.ucsc.edu/).

### Consent

Written informed consent was obtained from the patient for participation in this study and publication of this report and any accompanying images.
